# Automatic Detection and Classification of Focal Liver Lesions Based on Deep Convolutional Neural Networks: A Preliminary Study

**DOI:** 10.3389/fonc.2020.581210

**Published:** 2021-01-29

**Authors:** Jiarong Zhou, Wenzhe Wang, Biwen Lei, Wenhao Ge, Yu Huang, Linshi Zhang, Yingcai Yan, Dongkai Zhou, Yuan Ding, Jian Wu, Weilin Wang

**Affiliations:** ^1^Department of Hepatobiliary and Pancreatic Surgery, The Second Affiliated Hospital, Zhejiang University School of Medicine, Hangzhou, China; ^2^Key Laboratory of Precision Diagnosis and Treatment for Hepatobiliary and Pancreatic Tumor of Zhejiang Province, Hangzhou, China; ^3^College of Computer Science and Technology, Zhejiang University, Hangzhou, China; ^4^Clinical Medicine Innovation Center of Precision Diagnosis and Treatment for Hepatobiliary and Pancreatic Diseases of Zhejiang University, Hangzhou, China; ^5^Clinical Research Center of Hepatobiliary and Pancreatic Diseases of Zhejiang Province, Hangzhou, China; ^6^Research Center of Diagnosis and Treatment Technology for Hepatocellular Carcinoma of Zhejiang Province, Hangzhou, China

**Keywords:** deep learning, focal liver lesions, detection, classification, computed tomography

## Abstract

With the increasing daily workload of physicians, computer-aided diagnosis (CAD) systems based on deep learning play an increasingly important role in pattern recognition of diagnostic medical images. In this paper, we propose a framework based on hierarchical convolutional neural networks (CNNs) for automatic detection and classification of focal liver lesions (FLLs) in multi-phasic computed tomography (CT). A total of 616 nodules, composed of three types of malignant lesions (hepatocellular carcinoma, intrahepatic cholangiocarcinoma, and metastasis) and benign lesions (hemangioma, focal nodular hyperplasia, and cyst), were randomly divided into training and test sets at an approximate ratio of 3:1. To evaluate the performance of our model, other commonly adopted CNN models and two physicians were included for comparison. Our model achieved the best results to detect FLLs, with an average test precision of 82.8%, recall of 93.4%, and F1-score of 87.8%. Our model initially classified FLLs into malignant and benign and then classified them into more detailed classes. For the binary and six-class classification, our model achieved average accuracy results of 82.5 and73.4%, respectively, which were better than the other three classification neural networks. Interestingly, the classification performance of the model was placed between a junior physician and a senior physician. Overall, this preliminary study demonstrates that our proposed multi-modality and multi-scale CNN structure can locate and classify FLLs accurately in a limited dataset, and would help inexperienced physicians to reach a diagnosis in clinical practice.

## Introduction

Liver cancer, which is one of the most malignant types, represents the second-highest leading cause of cancer death in men worldwide, with a 5-year survival rate of less than 18% (1, 2). Hepatocellular carcinoma (HCC) is the most common type of liver cancer, accounting for approximately 90% of the total (3). Apart from HCC, several other types of lesions also frequently occur in the liver, including malignant lesions such as intrahepatic cholangiocarcinoma (ICC), metastases of malignant tumors from other tissues, benign lesions such as hemangioma (HEM), focal nodular hyperplasia (FNH), and cysts. Therefore, early detection and precise classification of focal liver lesions (FLLs) are particularly important for subsequent effective treatment.

Diagnostic radiographic imaging such as dynamic contrast-enhanced computed tomography (CT) provides useful information for the differential diagnosis of the aforementioned FLLs (4, 5). However, there are two obvious deficiencies. First, the evaluation of these images is usually subjective, as it is mostly dependent on the physicians’ experience. Second, physicians have to decide on the presence of lesions based on an exhaustive examination of slice-by-slice in CT images, which is very time-consuming. A recent study reported that, in some cases, radiologists have to read CT images at a speed of every 3–4 s per image during an 8 h workday to meet their workload demands (6). Under such constraints, errors are inevitable because radiology relies on visual perception and decision-making in cases of uncertainty (7, 8). The automatic detection and classification of lesions in diagnostic images using a computer-aided diagnosis (CAD) system has been developed to overcome these issues.

Deep learning (DL), an emerging branch of machine learning, can automatically extract and learn features from data in a complex nonlinear procedure based on a neural network structure (8, 9). At present, numerous studies have shown that DL, particularly convolutional neural network (CNN) models, can be used for pattern recognition of various organs and tissues, such as lung (10), renal (11), breast (12), retina (13), and skin (14) in diagnostic medical images, and can achieve satisfactory results. Two-dimension (2D) CNN models used on each CT or magnetic resonance imaging (MRI) slice have become mainstream to detect or classify FLLs (15–18). However, there are two disadvantages. First, they ignore the problem of the spatial discontinuity between the slices (19), leading to detection failure in some slices and diagnostic errors. Second, they focus on either the detection task alone or the classification task alone, ignoring the interconnection between them.

**Figure 1:**
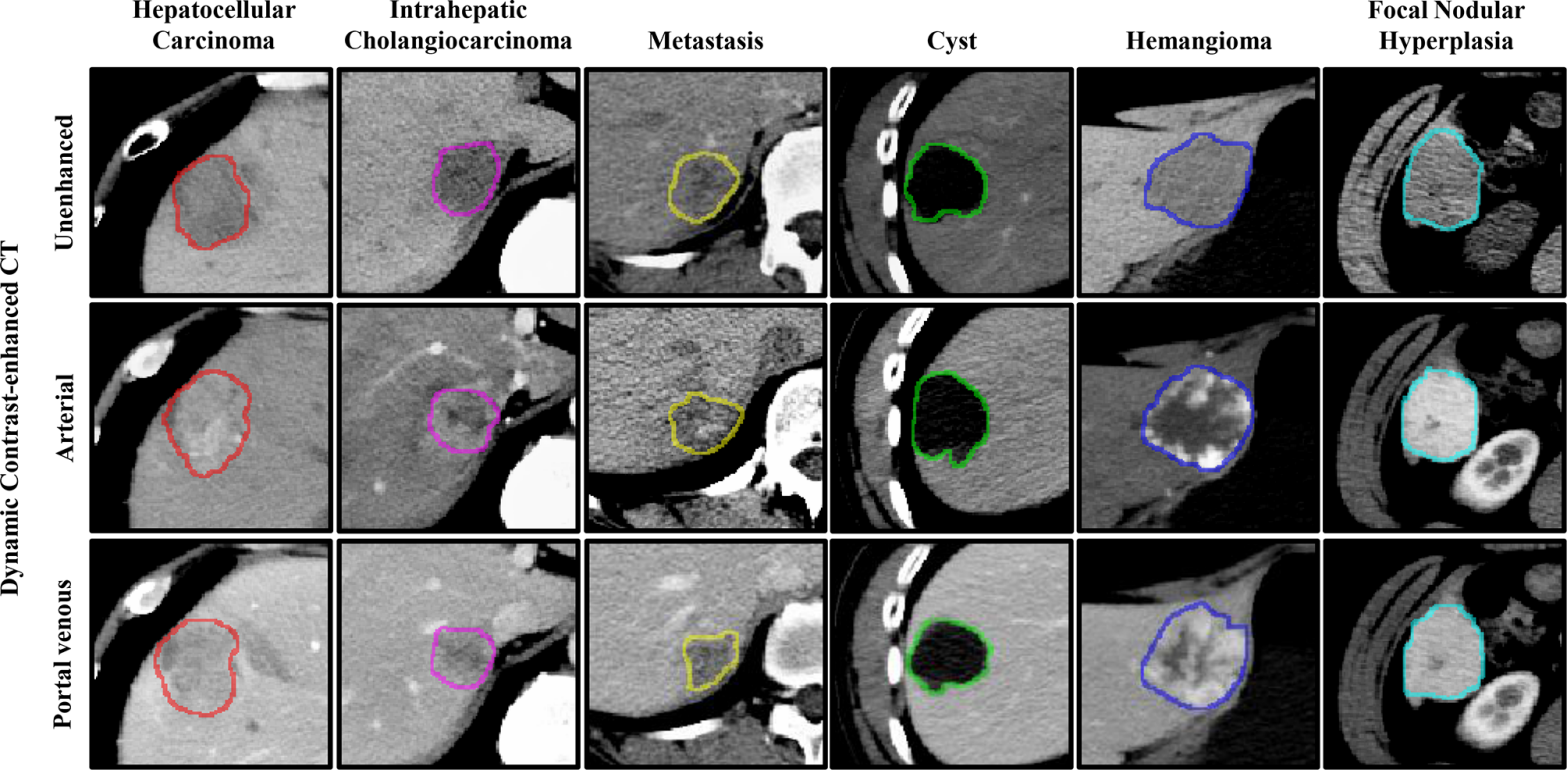
Representative images of focal liver lesions (FLLs) in different phases of dynamic contrast-enhanced computed tomography (CT). The contours represent the manually labeled boundary of the nodules.

Therefore, three-dimension (3D) approaches may be more favorable, particularly for classification, as they can address the problem of discontinuity and can provide more detailed spatial and structural information of the lesions. Much research has focused on the detection and classification of lung nodules using 3D strategies (10, 20–22). However, 3D approaches are not perfect for the detection of FLLs in a limited dataset, particularly 3D deep CNNs (19). The detection of FLLs is more difficult due to the variation in the six types of FLLs present in the background (e.g., blood vessels and bile ducts) and inherent characteristics (e.g., size, texture, density, and scale), as shown in **Figure 1**. Besides, compared to 2D CNNs, 3D CNNs usually suffer from parameter explosion and slow inference speed due to the additional dimension (23). Thus, approaches that lie somewhere between 2D and 3D (i.e., 2.5D) may be a preferred option to detect FLLs.

In this study, we developed a strategy based on a multi-modality and multi-scale CNN structure, composed of three 2.5D Faster R-CNN w/FPN and one 3D ResNet-18, for the automatic detection and classification of FLLs in three-phases (unenhanced, arterial, and portal venous phases) of CT images, respectively. Through a series of comparison experiments, we show that our established model performed better than other commonly adopted strategies, and would help physicians to diagnose FLLs in their daily practice.

## Materials and Methods

### Dataset

The multi-phasic CT data used in this study were collected from January 2016 to December 2018 *via* the picture archiving and communication system (PACS) in our institution. After searching and excluding non-FLL cases, 435 patients with a total of 616 liver lesions were enrolled in this study. The inclusion criteria were as follows: at least one FLL appeared in the CT image; malignant tumors had corresponding pathological diagnosis results; patients had not undergone any disease-related treatment before CT inspection, including transarterial chemoembolization therapy, surgery, radiofrequency abolition, and systemic chemotherapy; image quality was high and clear.

Among the 616 liver lesions, 280 (109 HCCs, 95 ICCs, and 76 metastases) were confirmed to be malignant tumors by tissue biopsy or postoperative pathology, and the remaining 336 (120 HEMs, 101 FNHs, and 115 cysts) were diagnosed as benign nodules through pathological analyses or a combination of typical image performance and clinical data. This retrospective study was approved by the institutional review board of the Second Affiliated Hospital, Zhejiang University School of Medicine and the need for patient informed consent was waived.

### CT Image Acquisition

Dynamic contrast-enhanced CT scanning was performed using three manufacturer’s CT models, i.e., SOMATOM Definition AS (Siemens), Brilliance iCT 256 (Philips), and Optima CT540 (GE Medical system), with the following parameters: tube voltage, 120 kVp; tube current, 250–600 mAs; pitch spacing 0.5–1 mm; and single collimation width, 0.625–1.25 mm. Three phasic images from unenhanced, arterial, and portal venous phases were enrolled in this study. During the enhancement phases, the concentration of contrast agents was determined by the patients’ body weight, with the criterion of 300 mg/mL iodine for patients weighing less than 60 kg, and 350 mg/mL iodine for those weighing more than 60 kg. In general, a total volume of 80–100 mL contrast material was injected into the patients at a rate of 3–4 mL/s with the use of a power injector *via* an 18- or 20-gauge cannula in the antecubital vein.

The scanning time of the arterial and portal venous phases was determined by the dose and injection rate of the contrast agents. In our institution, the scanning time of the arterial phase lasted ~20 s, during which the enhancement of the abdominal aorta reached its peak, and the CT enhancement value of the liver parenchyma was less than 10 HU. The period ended when the enhancement of the aorta decreased slightly, and the enhancement value of the liver parenchyma was between 10–20 HU. The scanning time of the portal venous phase usually started at 60 s after injection of the contrast medium. The slice thicknesses of the unenhanced and contrast-enhanced images were 5 mm and 0.8–5 mm, respectively.

### Data Annotation and Processing

To maximize the quality and resolution of CT images, all of the images were exported from our institution’s PACS and stored in a Digital Imaging and Communications in Medicine (DICOM) format. Medical image processing software (ITK-SNAP (24)), was used to label the regions of interest (ROIs) of the liver lesions in each CT image slice by two physicians (with 3 years’ experience in abdominal CT reporting), and then these ROIs were confirmed by another physician (with 30 years’ experience in abdominal CT reporting). Large blood vessels and bile ducts were excluded as much as possible from the region of the lesions. Among the 616 liver lesions, a quarter of nodules were randomly selected to make up the test set, and the remaining nodules formed the training set. Data augmentation is often used in the training set during deep learning to improve learning and extract features (25). In this study, each image was augmented randomly using the following five strategies: random flipping, random rotation, brightness transformation, Gaussian blur, and elastic transformation.

### Hierarchical Multi-Phase Lesion Detection and Classification Framework

**Figure 2:**
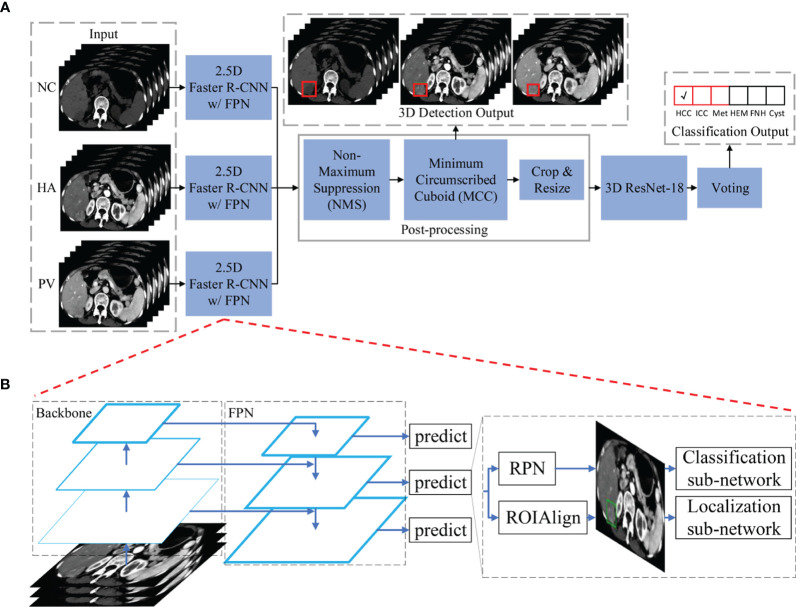
The main architecture of the hierarchical multi-phase lesion detection and classification framework. **(A)** The entire framework is constructed by three 2.5D detection networks (Faster R-CNN w/FPN) and one 3D classification network (ResNet-18). **(B)** The detailed architecture of the detection networks is composed of five components: a backbone convolutional network for basic feature learning and generating feature maps, a feature pyramid network (FPN) to exploit the inherent multi-scale, pyramidal hierarchy of the backbone network to construct feature pyramids with marginal extra cost, a region proposal network (RPN) that outputs a set of rectangular object proposals with objectness scores, and two sub-networks that produce bounding box regression and foreground/background classification. Abbreviations: NC, non-contrast; HA, hepatic artery; PV, portal venous; HCC, hepatocellular carcinoma; ICC, intrahepatic cholangiocarcinoma; Met, metastasis; HEM, hemangioma; FNH, focal nodular hyperplasia.

The main architecture of the framework used in this study is shown in **Figure 2A**. Our framework contains three 2.5D detection networks (Faster R-CNN w/FPN (26)) and one 3D classification network (ResNet-18 (25)) which were used for feature extraction and learning. Following the detection and classification networks, post-processing and voting modules were applied, respectively, to process the received information further and to generate detection and classification outputs.

### Detection Networks

Three detection networks were utilized for lesion localization in three phases of CT images, respectively. All three detection networks shared the same architecture because the scale of each type of lesion in different phases was almost identical, and differences in the six lesion types in each phase were similar. The first reason ensures that the detection networks can utilize the same receptive field and bounding box settings during network training and inference, and the second reason requires networks with similar learning abilities. We utilized a modified Faster R-CNN w/FPN to detect the six types of FLLs in three phases. The network was composed of five components: a backbone convolutional network for basic feature learning and generating feature maps, a feature pyramid network (FPN) to exploit the inherent multi-scale, pyramidal hierarchy of the backbone network to construct feature pyramids with marginal extra cost, a region proposal network (RPN) that outputs a set of rectangular object proposals with objectness scores, and two sub-networks that produce bounding box regression and foreground/background classification. A detailed diagram of our detection network is shown in **Figure 2B**. As described in the *Introduction* section, a trade-off between 2D and 3D is better for deep CNN training. We defined our 2.5D strategy as follows: To input each of the detection networks, each slice in a single CT image was concatenated with the two nearest neighbor slices (if these existed). The input size of the detection networks was 512×512×3. The ground truth label of one input was the annotation of the lesions in the center slice. For our task, we utilized ResNet-101 as proposed in a previous study (25), as our backbone network. Instead of using ROIPool (27) to extract small feature maps (e.g., 7×7) from each ROI (26, 28), ROIAlign (29) was utilized to obtain better results. Nine anchors with three aspect ratios [(1.5:1, 1:1, 1:1.5)] at three scales [(1, 2^1/3^, 2^2/3^)] were utilized on each of the pyramid levels. Each anchor was assigned with a 2-dimensional one-hot vector representing its class (background, foreground) and a 4-dimensional vector representing the coordinates of the upper left and lower right corners of the rectangular box surrounding the objects.

The classification sub-networks were supervised by binary cross-entropy (BCE) loss and the localization networks were supervised by smooth L1 loss (28). Because the backbone networks were utilized for basic feature learning and were deep, to train them from scratch would be time-consuming. Thus, we initialized parameters in the backbone networks with pre-trained parameters on the ImageNet dataset (30) before network training, and fine-tuned them with CT images afterwards. This kind of transfer learning strategy can effectively improve the representativeness of models (31). Parameters outside the backbone networks were randomly initialized by drawing weights from a zero-mean Gaussian distribution with a standard deviation of 0.01. The networks were trained for 200 epochs with a batch size of eight and the Adam optimizer (32). The learning rate started at 1e-3 and was decreased at the 40th, 80th, and 120th epochs by multiplying by 0.1.

After acquiring the detection outputs, we applied non-maximum suppression (NMS) to the proposal regions based on their classification scores to reduce redundancy (28). The Intersection-over-Union (IoU) threshold of the NMS was set to 0.5. Finally, we collected all of the localization results in each CT image and generated a minimum circumscribed cuboid (MCC) for each lesion detected.

### Classification Network

Because the outputs of detection networks only classify regions into foreground and background, we required a classification network to classify the foreground into detailed FLL types. We utilized a 3D version of ResNet-18 by modifying all of the 2D operations in a previous study (25) to 3D. It should be noted that because the lesions detected in the detection networks were not the same size, the input size of the CNNs during training should remain the same if the batch size is larger than 1. We resized all of the regions to 64×64×64 as the input for 3D ResNet-18. However, in real-world applications, not all three phases of the CT images are always captured. It may not be sufficient to concatenate the three phases of the CT images as the input for 3D ResNet-18. For this reason, we input each phase of each case into the network successively and utilized a voting strategy that collected the classification scores of multiple phases and calculated the average classification results for each CT image.

The 3D ResNet-18 was supervised by cross-entropy loss. We randomly initialized the parameters by drawing weights from a zero-mean Gaussian distribution with a standard deviation of 0.01. The network was trained for 200 epochs with a batch size of eight and a stochastic gradient descent (SGD) optimizer with a momentum of 0.9 and weight decay le-4. The learning rate started from 1e-3 and was decreased at the 60th and 120th epochs by multiplying by 0.1.

### Observer Study Evaluation

To compare the performance between our framework and humans in the classification of FLLs, two physicians (with 3 and 8 years of experience in abdominal CT interpretation, respectively) were enrolled in the observer study. The physicians did not participate in the model construction and data processing. In addition, the histopathology and other clinical data of the FLLs were not available to them. Each physician independently classified the FLLs from the test dataset into two and six categories using three-phasic CT images (unenhanced, arterial, and portal venous phases).

### Statistical Analyses

Because our framework contained two types of outputs (detection and classification results), different metrics were used for their evaluation.

Precision, recall, and F1-score were utilized to evaluate whether the FLLs were detected using our detection networks, and IoU was utilized to evaluate the size of the detected regions compared to ground truth annotation. Using the ground truth acquired from the annotation by the physicians, we categorized the prediction of the detection networks as true-positive (TP), false-positive (FP), true-negative (TN), and false-negative (FN). We denoted positive as detecting the FLLs and negative as detecting the background. True and false correspond to the presence of- and the absence of a match with the annotation results, respectively. The precision, recall, and F1-score could then be calculated as follows:

$$Precision=\;\frac{TP}{TP\;+\;FP},\;Recall=\;\frac{TP}{TP\;+\;FN},\;\text{and}\;F1=\;\frac{2*\;Precision*Recall}{Precision+Recall}.$$

IoU is defined as follows:

$$IoU=\frac{detected\;regions\cap \;\;annotated\;regions}{detected\;regions\;\cup \;\;annotated\;regions}$$

Sensitivity, specificity, receiver operating characteristic (ROC) curves, area under the receiver operating characteristic curves (AUCs), and average accuracy were utilized to evaluate the performance of the classification results. For each FLL class *c*, we denoted positive as classifying FLLs into *c*, and negative as classifying FLLs into other classes. True and false corresponded to the presence of- and the absence of a match with the annotation results, respectively. Sensitivity was equal to recall as defined above, and specificity and accuracy were calculated as follows:

$$Specificity=\;\frac{TN}{TN\;+\;FP},\;Accuracy=\;\frac{TP\;+\;TN}{TP\;+\;FP\;+\;TN\;+\;FN}.$$

The trained framework was tested by inputting CT images in the test dataset to the three detection networks as inputs. Then the detection and classification outputs were obtained, and the above metrics were calculated by comparing the outputs with the ground truth annotation. Our framework was trained and tested five times to reduce the variance of the neural network training, and the averaged results are reported.

## Results

### Evaluation of FLL Detection

**Figure 3:**
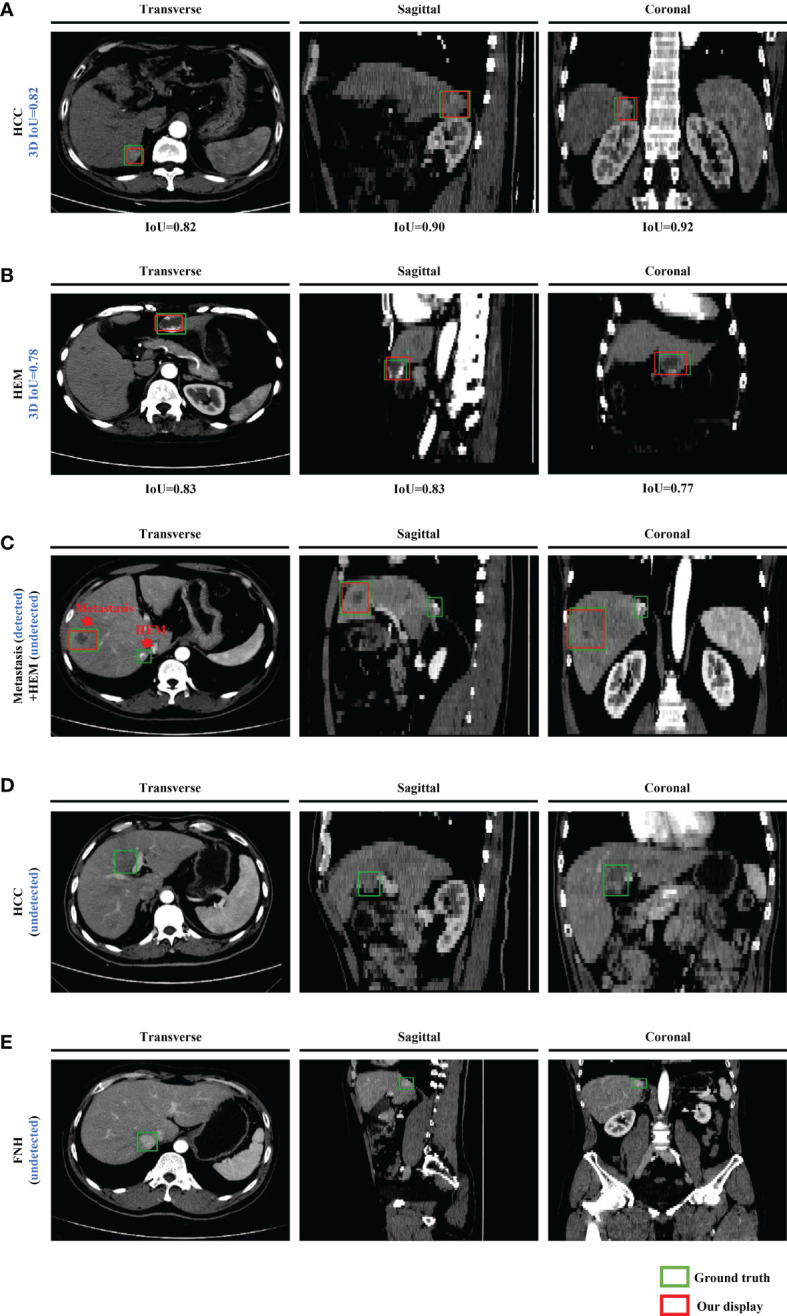
Representative images of the model-generated detection frames of lesions for five test cases in the transverse, sagittal, and coronal planes. Manually labeled and model-generated frames of **(A)** HCC, **(B)** HEM, **(C)** metastasis and HEM, **(D)** HCC, **(E)** FNH are shown in green and red, respectively.

After training, all FLLs in the test set were evaluated *via* our established detection network. We conducted five experiments using the same settings to reduce the variance in the training of the neural networks, and further evaluated the detection performance based on the metrics obtained from the test set. Representative images of the detection frames generated by the model for five test cases are presented in **Figure 3**. As shown in **Figure 3A**, an HCC lesion with a maximum diameter of 2.6 cm, was easily detected in 3D CT images. The 2D IoU values of the nodule in the transverse, sagittal, and coronal planes were 0.82, 0.90, and 0.92, respectively, and the 3D IoU value reached 0.82. For benign lesions, **Figure 3B** shows the detection result for a HEM, with a maximum diameter less than 3 cm. After analyses and calculation, the 3D IoU value of the lesion was 0.78. However, a small number of lesions with relatively small diameters or densities close to that of the surrounding liver parenchyma or blood vessels were easily overlooked and were ultimately not detected by our model (**Figures 3C–E**). In general, our framework achieved an average test precision of 82.8%, recall of 93.4%, F1-score of 87.8%, and a 3D IoU value of 34.8% (**Table 1**).

**Table 1:** Detection results of different networks in comparison experiments.

Approach	Post-process	Dimension	Precision (%)	Recall (%)	F1-score (%)	IoU (%)
RetinaNet (33)	No	2	70.3	89.2	78.6	27.8
		3	72.2	85.4	78.2	26.8
		2.5	71.1	89.8	79.4	29.1
	Yes	2	74.9	88.1	81.0	30.1
		3	78.0	81.8	79.9	29.3
		2.5	75.4	88.9	81.6	30.8
Faster R-CNN (28)	No	2	71.5	90.1	79.7	28.8
		3	72.8	86.0	78.9	27.9
		2.5	72.1	91.9	80.8	29.9
	Yes	2	77.4	88.6	82.6	31.5
		3	79.2	83.5	81.3	30.3
		2.5	77.8	90.1	83.5	32.3
**Ours**	**Yes**	**2.5**	**82.8**	**93.4**	**87.8**	**34.8**

CNN, convolutional neural network; IoU, intersection-over-union. The bold values represent the results obtained from our proposed model.

To evaluate the performance of our detection networks, we compared our results with RetinaNet, a commonly adopted detection network (33), and with the original Faster R-CNN (28). We also evaluated the importance of post-processing (the NMS module) and the performance of networks using different dimensions. We utilized the same network settings and hyper-parameters for all of the network training to ensure fair comparisons. For the 3D networks, the detection outputs were already cuboid, and so the MCC module was of no value and was not utilized under these conditions.

The results of comparison experiments are listed in **Table 1**. Faster R-CNN commonly outperformed RetinaNet. This may be because Faster R-CNN is a two-stage detection network, which makes it more complicated and time-consuming (34). In terms of the different dimensions in the same network structures, 2.5D networks commonly outperformed 2D networks, and 3D networks performed the worst. Furthermore, the networks commonly achieved better results with post-processing, indicating the importance of post-processing. Overall, 2.5D Faster R-CNN with post-processing performed better than all of the other experiments, and our modified 2.5D Faster R-CNN w/FPN achieved the best results. This suggests that our modified network structure is effective and would lead to improved FLL detection.

### Evaluation of FLL Classification

After the lesions were detected using our detection network, they were classified using the 3D ResNet-18 deep neural network. This was divided into two steps. We first classified FLLs into malignant (HCC, ICC, and metastasis) and benign (HEM, FNH, and cyst), after which they were further classified into detailed classes. The binary and six-class classification results were evaluated. To further assess the performance of our classification network, we compared the results with both the commonly adopted classification networks (2D Faster R-CNN (28), 3D VGG-16 (35) and the original 2D ResNet-18 (25)) and those of two physicians (a junior and senior physician with 3 and 8 years of experience in abdominal CT reporting, respectively).

**Figure 4:**
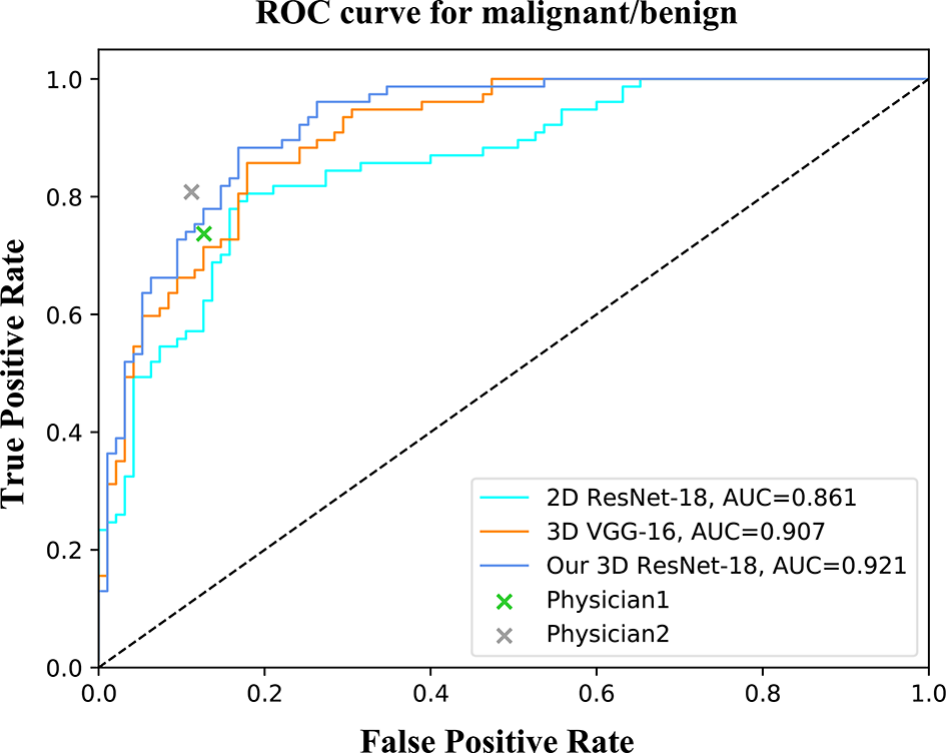
ROC curves of different networks for binary classification. The performance of physicians in the observer study is also shown in the graph.

For the binary classification, our model achieved an average classification accuracy of 82.5% with an average sensitivity of 76.6%, and specificity of 88.4% for malignant lesions, as well as an average sensitivity of 88.4% and specificity of 76.6% for benign lesions (**Table 2**). Through a series of comparison experiments, the performance of our 3D ResNet-18 model was better than the other three networks, and particularly the 2D networks (**Table 2**). In the observer study, the junior physician (physician 1) obtained slightly worse results than our model, where the overall accuracy was 80.4%, and the sensitivity and specificity of malignant lesions were 73.7 and 87.3%, respectively (**Table 2**). However, the classification results obtained from the senior (physician 2) were better than our model (**Table 2**). In addition, a ROC curve was generated by varying the probability threshold at which the model would classify a lesion as malignant (**Figure 4**). From that, our model achieved a better result with an AUC of 0.921 compared to 0.861 in 2D ResNet-18 and 0.907 in 3D VGG-16 models, respectively.

**Table 2:** Binary classification results of different networks and physicians.

Approach	Malignant		Benign		Average accuracy (%)
	Sen.	Spe.	Sen.	Spe.
	(%)	(%)	(%)	(%)
2D ResNet-18 (25)	70.1	82.0	82.0	70.1	76.2
2D Faster R-CNN (28)	65.6	78.9	78.9	65.6	72.7
3D VGG-16 (35)	74.8	86.2	86.2	74.8	81.1
Physician 1	73.7	87.3	87.3	73.7	80.4
Physician 2	80.8	88.8	88.8	80.8	84.6
**Our 3D ResNet-18**	**76.6**	**88.4**	**88.4**	**76.6**	**82.5**

“Sen.” is short for sensitivity, “Spe.” is short for specificity. “Physician 1” and “Physician 2” represent the junior and senior physicians, respectively. The bold values represent the results obtained from our proposed model.

**Figure 5:**
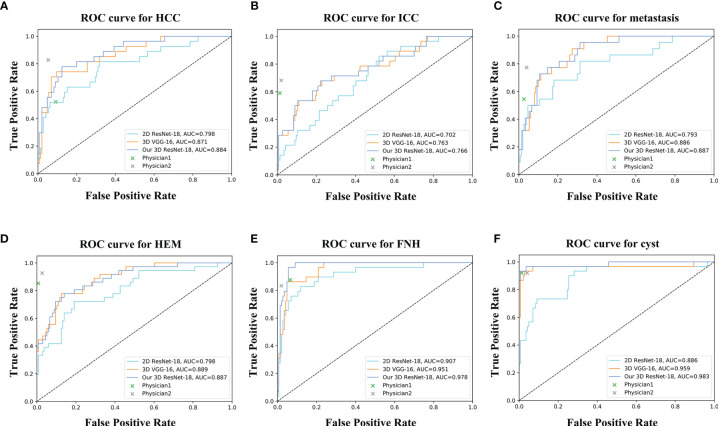
ROC curves of different networks for discriminating **(A)** HCC, **(B)** ICC, **(C)** metastasis, **(D)** HEM, **(E)** FNH, and **(F)** cyst. The performance of physicians in fine classification is also shown in the graph.

For the six-class classification, our model sensitivity ranged from 46.4% (for ICC) to 93.1% (for FNH), with specificity ranging from 91.9% (for HEM) to 98.6% (for cyst) (**Table 3**). As can be seen, the model’s performance in fine classification of benign lesions was higher than that of malignant lesions. This may be because malignant lesions are more similar in their inherent characteristics compared to benign lesions, and therefore, more samples are needed to improve the accuracy. As depicted in **Table 4**, our model achieved the best results with an average accuracy of 73.4% across the six lesion types compared to the other three networks. Moreover, the AUCs of each category ranged from 0.766 (for ICC) to 0.983 (for cyst) in our 3D ResNet-18 model, which outperformed the 2D ResNet-18 and 3D VGG-16 networks (**Figure 5**). Similar to the binary classification results, the performance of our model in the six-class classification was better than the junior physician but worse than the senior physician (**Table 4**). However, the total time taken for our model to detect and analyze each lesion was 2 ± 0.5 s, whereas it was 21.4 ± 9.2 s and 27.2 ± 9.3 s for the physicians, respectively (**Table 4**). This suggests that our model saves time and may help physicians with their diagnoses in daily clinical practice.

**Table 3:** Six-class classification results of different networks and physicians.

Approach	HCC		ICC		Metastasis		HEM		FNH		Cyst	
	Sen. (%)	Spe. (%)	Sen. (%)	Spe. (%)	Sen. (%)	Spe. (%)	Sen. (%)	Spe. (%)	Sen. (%)	Spe. (%)	Sen. (%)	Spe. (%)
2D ResNet-18 (25)	53.3	83.6	41.3	89.1	53.1	82.6	68.0	81.9	85.1	89.3	81.1	89.8
2D Faster R-CNN (28)	51.1	81.9	38.6	88.7	49.6	81.5	65.2	80.4	82.7	87.8	80.1	87.5
3D VGG-16 (35)	62.4	91.2	44.9	94.3	62.7	92.6	77.1	90.5	90.7	91.7	89.5	96.3
Physician 1	52.2	90.8	59.1	98.7	54.5	97.4	85.2	99.3	87.5	93.4	92.0	98.7
Physician 2	82.6	94.7	68.2	98.0	77.3	96.0	92.6	97.3	83.3	98.0	92.0	95.9
**Our 3D** **ResNet-18**	**63.0**	**93.1**	**46.4**	**95.8**	**63.6**	**93.3**	**77.8**	**91.9**	**93.1**	**95.1**	**90.0**	**98.6**

“Sen.” is short for sensitivity, “Spe.” is short for specificity. “Physician 1” and “Physician 2” represent the junior and senior physicians, respectively. The bold values represent the results obtained from our proposed model.

**Table 4:** Average accuracy and runtime of networks and physicians for six-class classification.

Approach	Average accuracy (%)	Runtime (S, mean ± SD)
2D ResNet-18 (25)	63.6	2.6 ± 0.4
2D Faster R-CNN (28)	62.2	1.8 ± 0.3
3D VGG-16 (35)	72.0	2.4 ± 0.3
Physician 1	72.7	21.4 ± 9.2
Physician 2	83.2	27.2 ± 9.3
**Our 3D ResNet-18**	**73.4**	**2.0 ± 0.5**

“Physician 1” and “Physician 2” represent the junior and senior physicians, respectively. SD, standard deviation. The bold values represent the results obtained from our proposed model.

## Discussion

Due to the increasing incidence of liver lesions and the increased workload of physicians, it is increasingly important to use noninvasive imaging analysis techniques to improve the accuracy of lesion detection and discrimination. In this study, we developed a CAD framework based on hierarchical CNN structures, which could simultaneously detect and classify six types of hepatic lesions in multi-phasic CT images, and achieve good results.

The advantages of DL systems are that they can constantly recognize, extract, and learn the different hierarchical features that are invisible to the human eyes. This assists physicians in detecting and classifying lesions, particularly those with atypical features in the images obtained. Previous studies have shown that CNN models can be used for the detection or classification of FLLs in single- or multi-phasic CT images by previously grouping those lesions into one to five categories (16, 17, 36–38). However, we investigated six types of hepatic lesions in our study, and three-phasic CT images from three scanners were utilized for the model training. This not only improved the ability of the model to detect lesions but also allowed the model to learn more features from dynamic contrast-enhanced CT images, thus facilitating subsequent nodule classification. Besides, it also demonstrated the robustness of our model using heterogeneous imaging sources from different CT scanners and acquisition protocol settings.

Chen et al. (39) demonstrated that both classification and localization of liver lesions could be handled using a dual-attention dilated residual network. However, we proposed three 2.5D Faster R-CNN w/FPN and one 3D ResNet-18 for the detection and classification tasks, respectively. We did not utilize the detection networks for both detection and classification even if they were able to classify the detected regions because the classification sub-networks in the detection networks were relatively shadowy, which makes the patterns within the six types of lesions difficult to learn. A relatively deep classification network would be required. On the other hand, our detection networks utilized a 2.5D strategy that divided each CT image into groups. The direct classification of each group generated different classes for a single lesion. To extract the features in the six types of lesions better, and to maintain the consistency of the lesion types, a 3D deep classification network was set up alone.

To address problems of spatial discontinuity in 2D CNN models (19) or parameter explosion and slower inference speed in 3D CNN models (23), we utilized a 2.5D strategy for lesion detection, which was a trade-off between 2D and 3D. Comparison experiments with another two CNN models showed the superiority of our modified model. Furthermore, we evaluated the importance of the model dimensions in the detection task. The 2.5D networks commonly outperformed the 2D networks, and the 3D networks performed the worst. The reason that 2D networks outperformed 3D networks is that more parameters were utilized for feature learning of each pixel/voxel in the CT images. Even if the 2D networks ignored the problem of the spatial discontinuity between the slices, they were still able to learn better within each slice. The 2.5D networks with post-processing performed the best because it utilized fewer parameters than the 2D networks for feature learning of each pixel/voxel, but the spatial discontinuity problem was alleviated.

The classification performance of our model was variable overall, with an average accuracy of 82.5 and 73.4% for binary and six-class classification, respectively. The finding was inferior to that of previous studies used a 2D CNN model (15, 18). However, our classification of liver lesions differed as it was based on the regions obtained from the previous detection networks, and we utilized the 3D CNN model. In addition, compared to 2D CNN structures, 3D CNNs utilize all of the spatial-temporal information contained in the 3D space, instead of just the local features, which assists with pattern recognition (40). This was validated in our experiments. Similar to the previous study (18), our model’s performance in fine classification of malignant nodules was lower than that of benign nodules, especially in ICC type. The reasons caused the lower accuracy of malignant lesions maybe as follows. Firstly, the variation of background (e.g., peritumoral enhancement and bile ducts dilation) and inherent characteristics (e.g., size, texture, density, and scale) in ICC CT imaging is more heterogeneous, and therefore, more samples should be included to improve the overall accuracy. Secondly, thinner layer-thickness of CT can improve the resolution and continuity of lesion region, which in turn helps to learn more detailed imaging features. Besides, introducing medical history and laboratory test results to CT imaging may be of better diagnostic value. To clinically evaluate the model, two physicians were enrolled in the observer study who classified the same cases in the test set. Interestingly, the classification performance of the model was placed between that of a junior physician and a senior physician. The diagnostic accuracy of the junior physician may increase or exceed that of the model if additional image sequences and clinical data are provided. However, for the model, limited data are an important factor that inhibits its performance.

While the results are promising and indicate the feasibility of our framework for the automatic and image-based detection and differentiation of malignant and benign FLLs, there are some limitations. First, overfitting is a critical issue attributed to the size of the dataset, particularly in complex models such as CNNs (9, 41). Because all of the data were collected from a single center, problems of bias and insufficient data were inevitable, resulting in a limited performance of the model during the training phase. To improve the applicability of the model and accelerate clinical transformation, additional training, and independent test sets from other institutions should be included. Second, we adopted some strategies to improve the models’ performance, such as an NMS module to reduce the redundant bounding boxes, and the detection and classification networks supervised by the ground truth labels separately to achieve better results during network training. However, for a cascaded and coupled design, it’s possible to degenerate the performance of classification network due to the error accumulation caused by the anterior detection networks. Third, lesions with a low incidence or those difficult to diagnose preoperatively were not enrolled in this study. Because the framework was effective for detecting and classifying six types of hepatic lesions commonly encountered in daily clinical practice, we will focus on uncommon types of liver lesions in the future. Besides, there was no corresponding histopathological assessment of all of the liver lesions. Because most benign lesions can be diagnosed by typical imaging findings or continuous follow-up evaluation, and do not involve subsequent surgical treatment, it is difficult to evaluate the histopathology for all lesions. For lesions without histopathological evaluations, ROIs were labeled by two physicians who analyzed all of the available imaging sequences and clinical data.

In summary, this preliminary study demonstrates that our proposed multi-modality and multi-scale CNN structure can locate and classify FLLs accurately, which could potentially be useful to help inexperienced physicians arrive at a diagnosis in daily clinical practice. With the increasing demand for radiological services, collaborative workflows that combine the experience and knowledge of physicians with DL-based CAD systems can provide more accurate disease diagnosis and higher quality patient care in a time- and labor-saving manner.

## Data Availability Statement

The raw data supporting the conclusions of this article will be made available by the authors, without undue reservation.

## Ethics Statement

The studies involving human participants were reviewed and approved by Independent institutional review boards of the Second Affiliated Hospital, Zhejiang University School of Medicine. Written informed consent for participation was not required for this study in accordance with the national legislation and the institutional requirements.

## Author Contributions

WLW, JW, JZ, and WZW designed the study. JZ and WZW participated in the majority of the experiments and wrote the manuscript. WZW and BL designed and constructed the models. YY searched and collected the dataset. WG, YH, and WLW performed the annotation and processing of the dataset. LZ and DZ took part in the observer study. YD and JW reviewed and revised the manuscript. All authors contributed to the article and approved the submitted version.

## Funding

This study was supported by the National Natural Science Foundation of China (grant numbers 81572307 and 81773096 to WLW) and National Natural Science Foundation of China (grant numbers 61672453 to JW).

## Conflict of Interest

The authors declare that the research was conducted in the absence of any commercial or financial relationships that could be construed as a potential conflict of interest.
